# Mapping tumour heterogeneity with pulsed 3D CEST MRI in non-enhancing glioma at 3 T

**DOI:** 10.1007/s10334-021-00911-6

**Published:** 2021-02-19

**Authors:** Esther A. H. Warnert, Tobias C. Wood, Fatih Incekara, Gareth J. Barker, Arnaud J. P. Vincent, Joost Schouten, Johan M. Kros, Martin van den Bent, Marion Smits, Juan A. Hernandez Tamames

**Affiliations:** 1 Department of Radiology and Nuclear Medicine, Erasmus MC, Rotterdam, NL the Netherlands; 2 Institute of Psychiatry, Psychology and Neuroscience, King’s College London, London, UK; 3 Department of Neurosurgery, Erasmus MC, Rotterdam, NL the Netherlands; 4 Department of Pathology, Erasmus MC, Rotterdam, NL the Netherlands; 5 Department of Neurology, Erasmus MC, Rotterdam, NL the Netherlands

**Keywords:** Non-enhancing glioma, CEST, APT, NOE

## Abstract

**Objective:**

Amide proton transfer (APT) weighted chemical exchange saturation transfer (CEST) imaging is increasingly used to investigate high-grade, enhancing brain tumours. Non-enhancing glioma is currently less studied, but shows heterogeneous pathophysiology with subtypes having equally poor prognosis as enhancing glioma. Here, we investigate the use of CEST MRI to best differentiate non-enhancing glioma from healthy tissue and image tumour heterogeneity.

**Materials & Methods:**

A 3D pulsed CEST sequence was applied at 3 Tesla with whole tumour coverage and 31 off-resonance frequencies (+6 to -6 ppm) in 18 patients with non-enhancing glioma. Magnetisation transfer ratio asymmetry (MTRasym) and Lorentzian difference (LD) maps at 3.5 ppm were compared for differentiation of tumour versus normal appearing white matter. Heterogeneity was mapped by calculating volume percentages of the tumour showing hyperintense APT-weighted signal.

**Results:**

LDamide gave greater effect sizes than MTRasym to differentiate non-enhancing glioma from normal appearing white matter. On average, 17.9 % ± 13.3 % (min–max: 2.4 %–54.5 %) of the tumour volume showed hyperintense LDamide in non-enhancing glioma.

**Conclusion:**

This works illustrates the need for whole tumour coverage to investigate heterogeneity in increased APT-weighted CEST signal in non-enhancing glioma. Future work should investigate whether targeting hyperintense LDamide regions for biopsies improves diagnosis of non-enhancing glioma.

**Supplementary Information:**

The online version of this article (10.1007/s10334-021-00911-6) contains supplementary material, which is available to authorized users.

## Introduction

Chemical exchange saturation transfer (CEST) is a technique to create magnetic resonance imaging (MRI) contrasts by selective targeting of labile protons in endogenous and mobile proteins. An emerging clinical application of CEST imaging is the assessment of glioma via amide proton transfer (APT) weighted CEST, which recently has shown promise for glioma grading [1, 2] and the differentiation between pseudoprogression and radiation necrosis [3].

Although patients with non-enhancing gliomas are often included in studies investigating APT-weighted CEST MRI, the main aim is often differentiation of glioma grade based on the classic histological classification of low to high-grade tumours, where high-grade tumours often show enhancement and form the majority of the studied population. The current body of literature indicates that lower grade gliomas are mostly isointense on amide proton transfer (APT)-weighted imaging, with potentially some areas of hyperintensity and that high-grade gliomas, i.e. glioblastomas, show increased APT-weighted CEST signal [1, 4]. However, in non-enhancing glioma, APT-weighted CEST could have an important role as these tumours can become very large and, like glioblastomas, have spatially varying pathophysiology and underlying molecular signatures [5]. The latter is of particular importance in light of the recently updated molecular classification of tumours as published by the World Health Organisation [6]. Whereas non-enhancing glioma used to be classified based on histology only, now two molecular parameters (IDH mutation and 1p/19q codeletion) are of interest that lead to three distinct classes of non-enhancing glioma [7]. These three classes differ widely in prognosis, ranging from a median overall survival of more than 10 years to just over 1 year, similar to enhancing glioblastoma (grade IV). Moreover, each of these classes warrants a different treatment regime, which stresses the need for accurate diagnosis.

Additionally, although the number of CEST research studies including multi-slice image acquisition schemes is increasing, previous investigations including non-enhancing glioma often use single-slice CEST acquisitions [4, 8, 9]. Because this excludes investigation of the whole tumour volume, care needs to be taken when interpreting the above results found about APT-weighted CEST signal in non-enhancing glioma. However, combining CEST preparations with a rapid 3D read-out has previously been shown to allow for the collection of multiple saturation offsets whilst covering the whole tumour volume in clinically feasible scan times [10, 11].

Here we use a 3D pulsed CEST sequence to explore APT-weighted signal in the whole tumour, specifically for non-enhancing glioma. We compare amide-weighted magnetisation transfer ratio asymmetry (MTR_asym_) and Lorentzian difference (LD) contrasts, CEST metrics widely used in the current literature. The former is commonly applied and is valued for its relative simplicity as it, in its essence, only requires measurement of the CEST effect when applying a B_1_ saturation pulse at two off resonance frequency shifts (3.5 ppm and − 3.5 ppm) in addition to a separate acquisition of multiple off-sets for B_0_-correction. The latter requires covering multiple off-resonance frequencies via the acquisiton of a full Z-spectrum to do Lorentzian fitting of the CEST signal. This analysis allows for separate investigation of signal contributions from amide protons at 3.5 ppm and nuclear Overhauser enhancement (NOE) at – 3.5 ppm. Note that in CEST experiments NOE contributes signal between − 1 and − 5 ppm, where aliphatic protons in mobile macromolecules are saturated. Via relayed-NOE this saturation is transferred to amide protons within the same molecule which will exchange with the free water pool [12]. Recently, NOE-weighted CEST signal has been shown to be correlated with prognosis [13, 14] and grading [15] of high-grade glioma. Therefore, we additionally investigate whether NOE-weighted CEST is of interest for non-enhancing glioma.

## Materials and methods

All images were acquired on a 3 T MRI scanner equipped with a 32-channel head coil (Discovery MR750, General Electric, Chicago, USA). All experiments were conducted in compliance with the declaration of Helsinki and under approval of the institutional ethics committee of the Erasmus MC (Rotterdam, NL), which is one out of 18 accredited medical research ethics committees in the Netherlands. Image analysis and statistical analysis were done with in-house written Matlab scripts (R2015b, The MathWorks, Natick, USA) and the freely available FMRIB Software Library (FSL 5.0.9, Oxford, UK).

### Patient information

Eighteen patients with newly diagnosed presumed low-grade glioma were recruited between March 2017 and March 2019. Patients were recruited as part of the Imaging Genomics study and were scanned at maximum 7 days before surgical resection or biopsy. Tumour samples were obtained and histopathologically examined by neuropathologists. Molecular classification of the 1p/19q co-deletion and IDH mutation status was performed as part of the diagnostic routine by molecular biologists with targeted Next-Generation Sequencing (NGS) panels using an Ion Torrent Personal Genome Machine or Ion S5XL (Thermo Fisher Scientific). Patient characteristics can be seen in Table 1.

**Tabel 1:** Patient characteristics

Patient	M/F	Age	Diagnosis (WHO 2016)	IDH mutation	1p/19q codeletion	Tumour volume (ml)	LD_amide_ (3.8 µT)	
							Hyperintense volume (ml)	Hyperintense volume (%)
P01	M	33	Oligodendroglioma – grade II	Yes	Yes	43.7	1.1	2.4
P02	F	57	Oligodendroglioma – grade II	Yes	Yes	177.6	41.2	23.2
P03	F	55	Oligodendroglioma – grade II	Yes	Yes	25.1	1.9	7.5
P04	M	35	Oligodendroglioma – grade II	Yes	Yes	227.1	57.0	25.1
P05	M	35	Oligodendroglioma – grade II	Yes	Yes	8.7	1.9	21.3
P06	M	31	Oligodendroglioma – grade II	Yes	Yes	16.9	1.1	6.4
P07	M	42	Oligodendroglioma – grade II	Yes	Yes	20.8	4.6	22.1
P08	M	39	Astrocytoma – grade II	Yes	No	80.0	28.1	35.1
P09	F	54	Astrocytoma – grade III	Yes	No	37.5	7.8	20.9
P10	F	46	Astrocytoma – grade III	Yes	No	62.6	21.0	33.6
P11	M	40	Astrocytoma – grade III	Yes	No	29.5	3.8	13.0
P12	F	24	Astrocytoma – grade III	Yes	No	79.7	4.1	5.2
P13	M	65	Glioblastoma—grade IV	No	No	12.8	7.0	54.5
P14	M	50	Anaplastic astrocytoma – grade III	No	No	13.8	1.8	13.4
P15	M	77	Glioblastoma – grade IV	No	No	20.2	1.2	5.8
P16	M	60	Glioblastoma – grade IV	No	–	40.5	5.4	13.3
P17	M	50	Glioblastoma – grade IV	No	No	78.6	9.1	11.5
P18	M	56	Glioblastoma – grade IV	No	No	13.5	1.1	8.0

### Image acquisition

CEST images were acquired with a 3D spoiled gradient echo with TR = 35.4 ms, TE = 6.9 ms, 12 slices, slice thickness = 5 mm, in-plane voxel size = 1.85 × 1.85 mm^2^, matrix = 128 × 128, acceleration factor = 4, similar to the pulsed CEST sequence used by Jones et al. [10]. Spectral-spatial excitation pulses were used to avoid fat artefacts in images near the water peak. [16] A Gaussian saturation pulse was played every TR with duration 20 ms (duty cycle 56.5%). Two CEST series were acquired at saturation powers B_1_ = 2.3 and 4.0 µT, and each series consisting of 31 frequency off-sets (± 6.0, ± 5.5, ± 5.0, ± 4.5, ± 4.0, ± 3.75, ± 3.5, ± 3.25, ± 3.0, ± 2.5, ± 2.0, ± 1.5, ± 1.0, ± 0.75, ± 0.5, ± 0.25, 0 ppm). Note that the saturation powers used are stated as the root mean square B_1_ across the saturation pulse. Two images were acquired without saturation pulses to obtain the equilibrium magnetisation (M_0_) for reference, bringing the total to 33 images acquired in $\sim$ 5 min. A B_1_ map was created by using a multi-slice 2D gradient echo sequence (TE = 12.8 ms, TR = 17 ms, flip angle = 10°) with voxel size and number of slices (1.85 × 1.85 × 5 mm^3^, matrix = 128 × 128x12) matched to the CEST sequence. T_1_-weighted (3D IR FSPGR, TE = 2. 1 ms, TR = 6.1 ms, voxel size = 1 × 1 × 0.5 mm^3^, field of view 256 mm, 352 slices) and T_2_-weighted FLAIR (3D spin echo read-out, voxel size = 1 × 1 × 1.6 mm^3^, matrix size = 224 × 224 × 264, TR/TE/TI = 6000 ms/112.9 ms/1893 ms) structural images were additionally acquired. Note that the 3D CEST acquisition was planned to cover the whole tumour, as indicated by the T_2_-FLAIR hyperintense area.

### Image analysis

Motion correction of the CEST image series was done by linear registration of each image within a series to the 6 ppm image and a cost function based on mutual information (*mcflirt*, within FSL v5.0.9, Oxford, UK), after which linear registration was used to register the CEST images to the magnitude of the B_1_-map. Z-spectra were calculated by dividing the images acquired with off-resonance saturation pulses by the average of the two M_0_ images. A Lorentzian curve was fitted to the Z-spectra using the data points with off-resonance frequency shifts of ± 6 ppm and those from − 1.75 to 1.75 ppm. This fit was first used for B_0_-correction, by shifting each spectrum by the frequency shift of the minimum value of the Lorentzian fit. Amide-weighted MTR_asym_ was calculated according to the methods described by Zhou et al. [17], using the B_0_-corrected Z(3.5 ppm) and Z(− 3.5 ppm). Lorentzian Difference (LD) analysis was used to obtain maps for LD_amide_ at 3.5 ppm and LD_NOE_ at − 3.5 ppm [18–20]. The MTR_asym_ and LD contrasts were calculated for both B_1_ saturation powers acquired. Contrast-based B_1_ correction was then carried out according to previously described methods, including the use of an artificial B_1_ = 0 µT [21], resulting in voxel-wise B_0_- & B_1_-corrected MTR_asym_, LD_amide_, and LD_NOE._ To avoid extrapolation in voxels with a B_1_ below the nominal value of B_1_ = 4.0 µT, in the remainder of the patient data the results are calculated for the images resulting from the B_1_-correction with B_1_ values of 2.5 and 3.8 µT.

Tumour regions of interest (ROI) were generated semi-automatically by delineating the hyperintense area on the T_2_-weighted FLAIR images using ITKSnap [22]. Note that no areas of necrosis were visually identifiable on the images acquired for the grade IV tumours (*N* = 6). The contralateral normal-appearing white matter (NAWM) ROI was generated by segmentation of white matter in the T_1_-weighted images (*fast,* within FSL v5.0.9, Oxford, UK) and a linear registration of this segmentation to the FLAIR image. NAWM was determined by using the white matter contralateral to the tumour in all slices that also included the tumour segmentation. Per patient, average tumour and NAWM MTR_asym_, LD_amide_, and LD_NOE_ were calculated.

To determine the extent of hyperintense amide-weighted CEST signal within the tumour per contrast and per patient, for each patient a threshold was determined as follows:

$$S_{thresh} > { }\overline{{S_{{amide,{ }NAWM}} }} + 2{*}\sigma_{NAWM} ,S_{thresh} > { }\overline{{S_{{amide,{ }NAWM}} }} + 2{*}\sigma_{NAWM} ,$$

where $\overline{{S_{{amide,{ }NAWM}} }}\overline{{S_{{amide,{ }NAWM}} }}$ is the average amide-weighted signal in NAWM and ${\sigma }_{NAWM}$ is the standard deviation of ${S}_{amide}$ in the NAWM. The percentage of voxels surpassing this threshold within the tumour ROI, as determined by dividing the numbers of voxels surpassing ${S}_{thresh}$ within the tumour ROI by the total number of voxels covering the T_2_-weighted FLAIR hyperintense tumour area, was calculated per slice and for the whole tumour for each patient. $S$ represents the four different amide-weighted contrasts calculated: MTR_asym_ and LD_amide_, both calculated for B_1_ is 2.5 and 3.8 µT.

### Statistical analysis

Mixed effects linear regression models were fitted to determine whether there were significant effects of the fixed effects *ROI* and *B*_*1*_ on the CEST contrasts generated in the patient data (MTR_asym_, LD_amide,_ and LD_NOE_). The *ROI* factor contained two levels (NAWM and Tumour) and *B*_*1*_ contained two levels (2.5 and 3.8 µT). To compare the different CEST contrasts for differentiating tumour tissue from NAWM effect sizes (Cohen’s *d*) were calculated for all three contrasts.

A mixed effects linear regression model was also used to investigate whether there was a significant effect of *contrast* (two levels, MTR_asym_ and LD_amide_) or *B*_*1*_ (two levels, 2.5 and 3.8 µT) on the volume percentage of the tumour showing hyperintense amide-weighted signal.

## Results

**Fig. 1:**
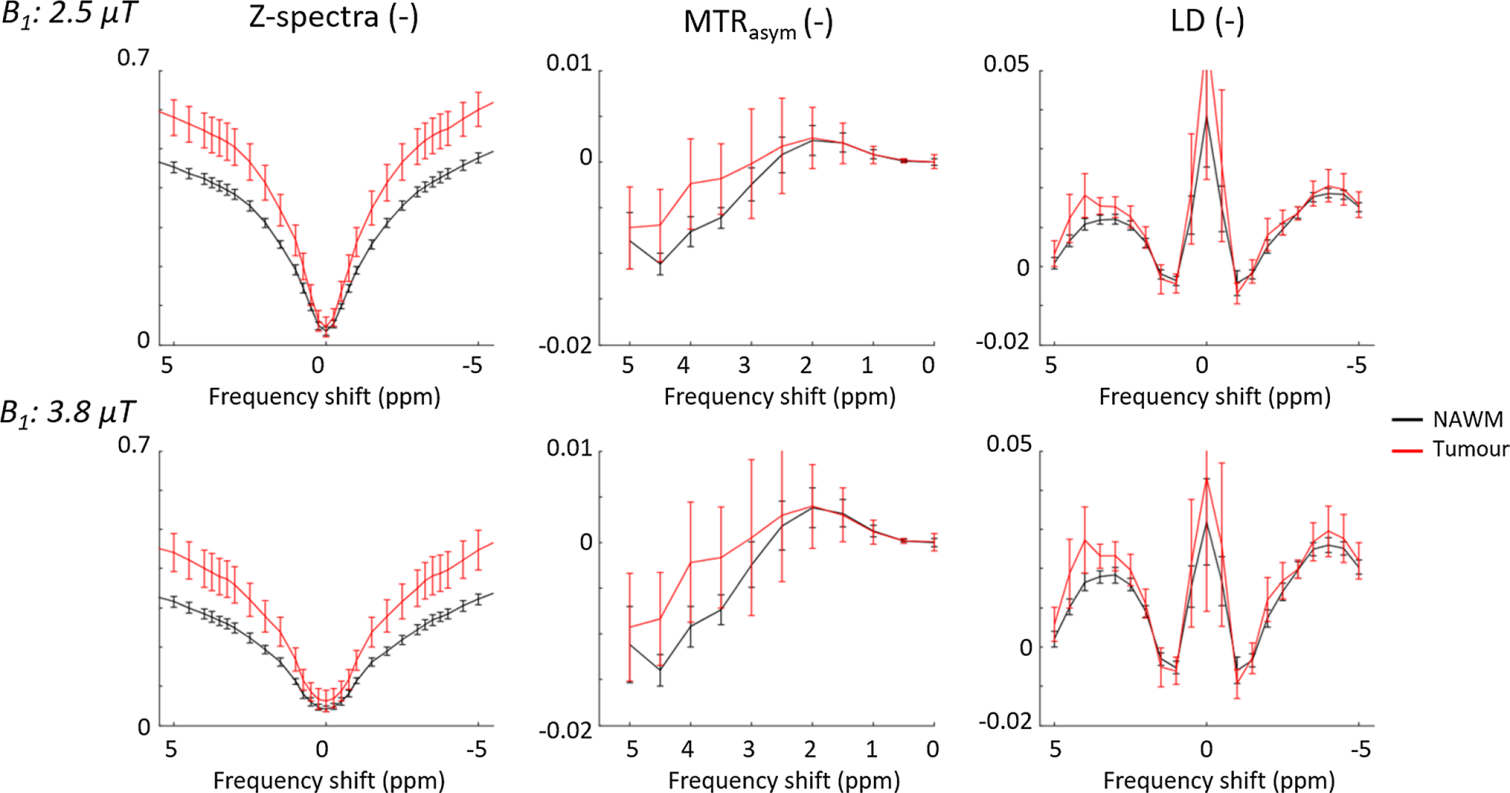
Z-spectra, MTR_asym_ and LD for NAWM and tumour ROI. The top row contains results for the B_1_ power of 2.5 µT, the bottom row contains results for the B_1_ power of 3.8 µT. Results are group averaged (N = 18) and errorbars represent the standard deviation across the group. The Z-spectra in the left column are corrected for B_0_ inhomogeneity, the MTR_asym_ and LD plots are additionally contrast B_1_ -corrected

Group averaged z-spectra, MTR_asym_ and LD are plotted in Fig. 1. Group averaged values for all three CEST contrasts per ROI are stated in Table 2. For MTR_asym_, LD_amide_, and LD_NOE_ the mixed-effects linear regression showed a significant effect of *ROI* (*p* < 0.05, Bonferroni corrected), indicating significant differences found between NAWM and tumour tissue. Post-hoc paired t-tests illustrate that only for MTR_asym_ and LD_amide_ there are significant increases in tumour tissue versus NAWM (Table 2).The largest effect size for differentiating the whole tumour ROI and NAWM was found for LD_amide_ when using a saturation power of 3.8 µT (Table 2).

**Table 2:** Averages and Cohen’s d effect size for CEST contrasts in non-enhancing glioma patients (N = 18)

	NAWM	Tumour	*p*-value Tumour vs NAWM	Cohen’s d Tumour vs NAWM
*B*_*1*_ = *2.5 µT*				
MTR_asym_	–0.006 ± 0.001	–0.002 ± 0.004	< 0.001*	1.1
LD_amide_	0.012 ± 0.001	0.015 ± 0.002	< 0.001*	1.6
LD_NOE_	0.017 ± 0.002	0.019 ± 0.003	0.200	0.3
*B*_*1*_ = *3.8 µT*				
MTR_asym_	− 0.007 ± 0.002	− 0.002 ± 0.006	< 0.001*	1.0
LD_amide_	0.018 ± 0.002	0.023 ± 0.003	< 0.001*	1.7
LD_NOE_	0.025 ± 0.002	0.027 ± 0.005	0.083	0.4

* NAWM and tumour value significantly different, *p* < 0.05, Bonferroni corrected

**Fig. 2:**
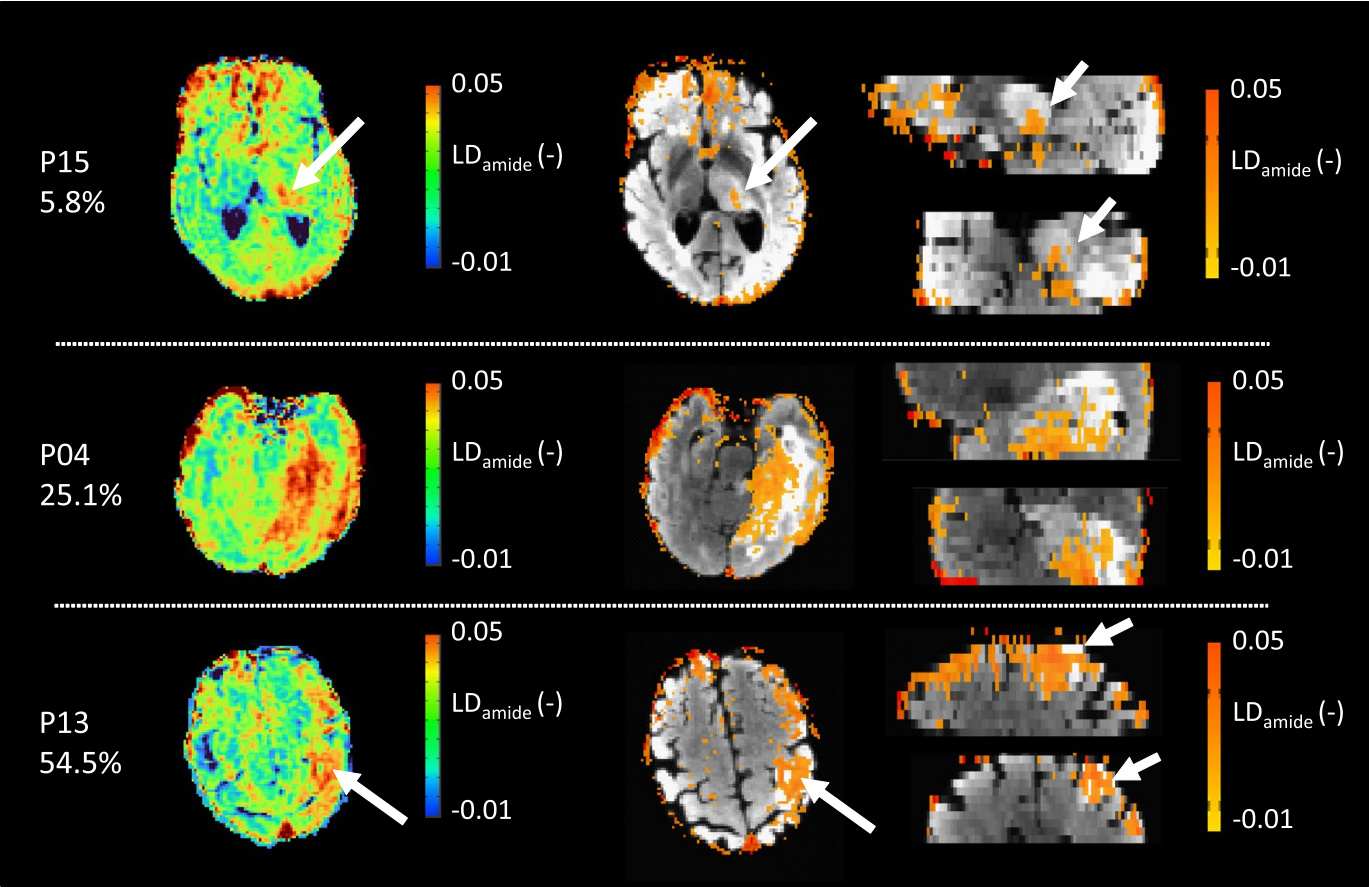
Examples of LD_amide_ images acquired for three different patients with a B_1_ saturation power of 3.8 µT. Each of these patients has a different volume percentage of voxels with an increased LD_amide_, which is stated below the patient number. The left colum shows the unthresholded LD_amide_ maps, followed by the map thresholded based on the NAWM LD_amide_. The thresholded images are overlaid on the T_2_-weighted FLAIR and axial (*left*), sagittal (*top*), and coronal slices (*bottom*) are presented

**Fig. 3:**
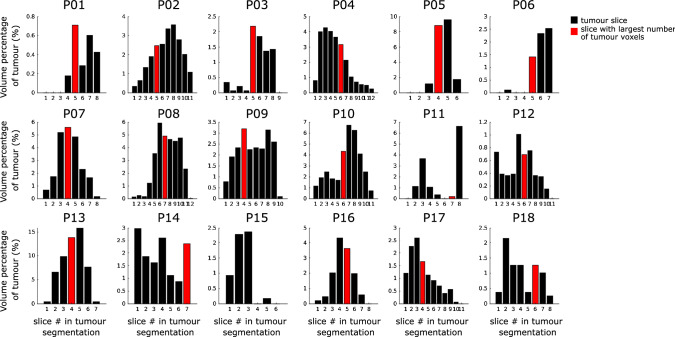
Volume percentage of tumour showing hyperintense LD_amide_ for the CEST acquisition with a B_1_ saturation power of 3.8 µT. Each plot contains the volume percentages per slice containing tumour tissue for one patient. Slice #1 is the most proximal slice. Note that each tumour has a different volume, reflected by different number of slices containing tumour tissue. The red bars indicate the slice with the largest number of tumour voxels. For P15 this is slice 4, which does not contain hyperintense LD_amide_ voxels

Examples of the images generated by thresholding the LD_amide_ CEST maps generated for B_1_ = 3.8 µT can be seen in Fig. 2. The hyperintense LD_amide_ voxels were heterogenously distributed across the tumours, as illustrated by Fig. 3. This figure illustrates that not necessarily all slices of the tumour contain hyperintense LD_amide_ signal. For example, in P01 the three most proximal slices of the tumour show no hyperintense LD_amide_ voxels. Figure 3 also illustrates that in 14 out of 18 patients the largest area of hyperintense LD_amide_ is not found in the slice with the largest tumour volume present. As an example, for P015 the slice with the largest amount of tumour voxels is slice 4, which contains no hyperintense LD_amide_ voxels.

**Fig. 4:**
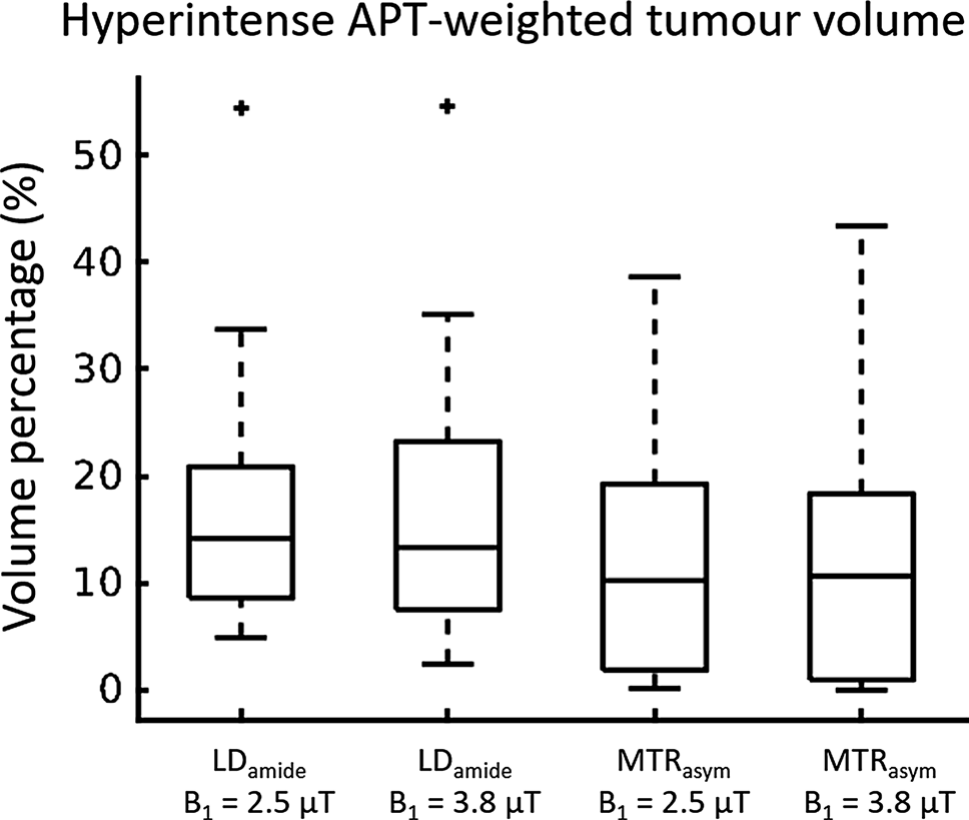
Boxplots of volume percentages showing hyperintense APT-weighted signal for MTR_asym_ and LD_amide_, for B_1_ = 2.5 µT and 3.8 µT (*N* = 18)*.* Although on average the LD_amide_ contrast leads to a slightly larger volume showing hyperintense signal in APT-weighted imaging, mixed-effect ANOVA did not show a significant effect of *contrast* on the volumes calculated (*p* = 0.045). Additionally, no significant effect of B_1_ power on the hyperintense volume percentage was found (*p* = 0.795)

On average, 17.9% ± 13.3% of the tumour volume showed hyperintense LD_amide_, which was based on a calculated *S*_*thresh*_ of 0.031 ± 0.003 (*N* = 18). The hyperintense tumour volume percentages found for LD_amide,B1=2.5 µT_, MTR_asym,B1=2.5 µT_, and MTR_asym,B1=3.8 µT_ were 17.1% ± 12.4%, 12.7% ± 11.7%, and 13.1 ± 13.0%, respectively (boxplots in Fig. 4). Although on average the LD_amide_ contrasts lead to a larger volume showing hyperintense signal in APT-weighted imaging, mixed-effects linear regressions did not show a significant effect of *contrast* on the volumes calculated (*p* = 0.045, which is larger than the Bonferroni corrected p-value threshold). Additionally, no significant effect of *B*_*1*_* power* on the hyperintense volume percentage was found (mixed-effects linear regression, *p* = 0.795).

## Discussion

We investigated APT-weighted CEST MRI in non-enhancing glioma and showed that Lorentzian difference analyses are preferred in these type of tumours over the use of MTR_asym_ calculations since the largest effect size to differentiate tumour from NAWM was found for LD_amide_. Another important finding of this study is that, despite the majority of the non-enhancing glioma volume showing isointense signal amide-weighted CEST images, on average approximately 18% of the total volume of the T_2_-FLAIR hyperintense area showed hyperintensity on LD_amide_ images. The large area of isointense signal within these tumours is as expected, as earlier work in which non-enhancing glioma is included in patient populations has also found largely isointense APT-weighted CEST contrast [1, 4]. However, this work in which whole tumour coverage of non-enhancing glioma is done indicates that there are areas with hyperintense APT-weighted signal. This finding has two important implications: (1) it illustrates the spatial heterogeneity of pathophysiology within non-enhancing glioma and (2) stresses the need for covering the whole tumour when acquiring APT-weighted CEST images.

In particular in light of the spatial heterogeneity in pathophysiology and molecular signatures in non-enhancing glioma [5], the regions with hyperintense amide-weighted CEST signal may be areas where aggressive tumour tissue is present. This is an hypothesis strengthened by the potential sensitivity of APT-weighted CEST to protein build-up during cell proliferation, as for instance shown by Togao et al.[8] and Jiang et al.[23, 24] in a correlation between MTR_asym_ and the Ki-67 labelling index, a histopathological marker of cell proliferation, in patients with low- and high-grade glioma. In a pre-clinical study by Yan et al. both total cytosolic protein content and APT-weighted CEST signal were increased in glioma compared to healthy tissue [25]. These previous studies indicate the potential clinical relevance of increased APT-weighted signal in locating active tumour tissue, an aspect that is of importance in future work to target the most aggressive area of a tumour for accurate diagnosis.

Note that, in addition to cell proliferation, it is well known that other sources of CEST signal exist that can contribute to differences in signal between tumour and healthy tissue. We find that the use of a B_1_ saturation power of 3.8 µT gave a stronger effect size than a B_1_ of 2.5 µT for separating tumour from NAWM when using LD_amide_. Note that, based on the theoretical optimal sensitivity of CEST MRI to amide protons when using a lower B_1_ saturation power (~ 1 µT [10]), this finding corroborates that there must be other sources (in part) responsible for differentiation of glioma and NAWM in our study. Based on previous studies, these other sources include the following: (i) a non-linear relationship between B_1_ saturation power and the ratio of T_1_-water relaxation times and overall water content, with the latter parameters (T_1_ of water and water content) likely to be increased in the tumour compared to NAWM [25], (ii) a decrease in magnetisation transfer from semisolid macromolecules in tumour compared NAWM [26], in particular, if increased B_1_ saturation power increases magnetisation transfer effects in NAWM in a stronger manner than in the tumour, (iii) contributions to the CEST signal from fast exchanging amine protons resonating around 2–3 ppm because of the relatively high B_1_ saturation pulse used here [27].

Although our results indicate that differences in APT-weighted CEST are found in non-enhancing glioma as well, it is thus clear that the origin of increased amide-weighted CEST contrasts in non-enhancing glioma is still to be investigated. This should first be done with an extensive MR imaging protocol, including quantification of T_1_, high-resolution structural imaging (pre- & post-contrast T_1_, T_2_-weighted FLAIR), and a CEST acquisition that allows for separation of CEST signal from amide protons, NOE and MT effects. Such a CEST acquisition could be designed to be as selective to amide protons as possible, for instance as done by Zaiss et al.[26] at ultrahigh field (9.4 T), with low B_1_ saturation power and the acquisition of a full Z-spectrum such that multi-pool Lorentzian fitting can be used to separate different CEST signal sources. To assess the extent to which amide protons are contributing to the differences in APT-weighted CEST imaging should be followed by targeted biopsies of tumour tissue for ex vivo analysis of the local environment with proteomics analysis [25, 28], rather than cell proliferation indices, that at minimum include measurement of cytosolic protein content and potentially further investigate specific mobile proteins and semisolid macromolecules contributing to the CEST signal.

Second, if the hyperintensities in amide-weighted CEST MRI are highlighting more aggressive tumour tissue it is important to not miss these regions when imaging non-enhancing glioma, stressing the need for having a CEST acquisition that covers the whole tumour volume. Note that although this may seem to go against the finding of Sakata et al. [2], who showed that for differentiation of low- and high-grade tumours single slice acquisition worked equally well as multi-slice acquisitions, the example of P15 in this work illustrates that hyperintense APT-weighted signal may be missed if a single slice is imaged. However, note that the work by Sakata et al. was conducted before the updated WHO tumour classification of 2016 in which the molecular diagnosis became important for grading. Moreover, advances in image acquisition for CEST MRI have enabled rapid measurement of full Z-spectra for multi-slice volumes in clinically feasible scan time, e.g. [10, 11]. Therefore, it is recommended in future work investigating CEST MRI for imaging diagnostics in non-enhancing glioma to use full tumour coverage as much as possible. This would for instance aid future research investigating whether amide-weighted CEST MRI can be used to direct biopsies for accurate non-enhancing glioma diagnosis in light of the recent WHO classification or improve the use of CEST MRI for differentiation of tumour progression and radiation necrosis in treatment follow-up in glioma patients.

Note that here we found that LD_amide_ results in a larger effect size than MTR_asym_ to differentiate non-enhancing glioma tissue from NAWM, which may be caused by finding no significant difference for LD_NOE_ between non-enhancing glioma and NAWM. This latter finding may be as expected, as previous studies on high-grade, enhancing gliomas showing that LD_NOE_ correlated with prognosis [13, 14] and grading [15], all done at 7 T, show that stronger decreases in LD_NOE_ compared to NAWM correlate to worse prognosis/grade in enhancing glioma, with limited changes in LD_NOE_ for tumours with better outcome. Moreover, at 3 T and in 11 high-grade glioma patients, Heo et al.[29] report only slight hypointensity in LD_NOE_ in tumour tissue. Extrapolating these results to this study in which we only include non-enhancing glioma may suggest that not finding a significant LD_NOE_ effect in the current study is plausible. Note that, as a consequence of finding limited effects of LD_NOE_, the largest effect size in separating NAWM from non-enhancing glioma was found for LD_amide_ in the current study, with MTR_asym_ being affected by both amide- and NOE-weighted signals within the tumour and hence the noise in MTR_asym_ being affected by CEST signal at both 3.5 and − 3.5 ppm, illustrated by the ratio of the standard deviations compared to the group averaged values being larger for MTR_asym_ (Table 2). Additionally, using MTR_asym_ there was a trend to find smaller hyperintense tumour volumes than using LD_amide_ maps. This suggests that with using MTR_asym_ regions of increased amide-weighted signal within the tumour may be missed. Although we do acknowledge that these findings at this stage are specific to the set-up as used here and an increased SNR for MTR_asym_ APT-weighted images, as can be obtained by increasing the number of acquisitions on and around ± 3.5 ppm [30], can lead to increased sensitivity to areas of increased APT-weighted signal for asymmetry analysis. Future work is required to further investigate whether this finding can be confirmed for non-enhancing glioma with a set-up optimised for MTR_asym_ analysis.

### Limitations

The number of included patients is likely too small for thorough investigation of using amide-weighted imaging for classification of non-enhancing glioma. Analyses to investigate differences between the three different tumour types, as well as IDH-mutation (*N* = 12) vs IDH-wildtype (*N* = 6) tumours were performed but resulted in no significant differences for the CEST contrasts as well as the size of the hyperintense volumes (results in Supplementary information). However, note that Paech et al. [13] and Jiang et al. [9] showed that amide proton weighted CEST signal has predictive value in assessing IDH mutation status, allowing for differentiation of low- and high-grade gliomas based on CEST imaging alone, potentially even within non-enhancing glioma solely. Future work is required to corroborate those findings.

B_0_-correction was carried out with the CEST data itself, rather than acquiring an external map with a low B_1_ saturation power [31]. This means that the B_0_ correction here may be affected by hydroxyl protons, resonating close to the water peak (~ 0.9 ppm [12]) causing potential inaccuracies. However, in the B_0_-correction performed points further than 1 ppm away from the water peak were excluded and with visual inspection of the Z-spectra for B_1_ is 2.5 μT (Fig. 2) it is not likely that B_0_-correction is affected by widening of the peak around the OH-peak. Note that the wider peak at the largest B_1_ used suggests that B_0_-correction for this acquisition may be contaminated by hydroxyl protons resonating near 0.9 ppm. This effectively means that the B_0_- correction at high B_1_ saturation power is overestimated and a larger shift towards the upfield frequencies is applied than strictly required. Effectively this would lead to underestimation of the APT-weighted signal at 3.5 ppm. The true extent to which this will affect the results in this work is hard to gauge. It is recommended in future work to use a separate acquisition with low B_1_ saturation power for B_0_-correction.

A steady-state CEST sequence is used in this work, as at the time of initiating this study it was deemed the most appropriate for rapid acquisition of whole tumour volume at our institute. However, there are alternatives now with long saturation blocks prior to image read-out that may result in higher signal-to-noise ratios for the CEST contrast images [11]. Future work, therefore, includes the investigation of heterogeneity in APT-weighted CEST signal in non-enhancing glioma with longer pre-saturation blocks.

### Conclusions

This study illustrates that 3D pulsed CEST imaging allows for measuring heterogeneity in amide-weighted CEST signal in non-enhancing glioma. Based on the results in this study we recommend to use CEST acquisitions that cover the whole tumour volume for assessment of non-enhancing glioma, to not miss areas of hyperintense APT-weighted signal which may be small within these tumours. Future work includes investigation of the cause for increased LD_amide_, which is a step towards the application of APT-weighted CEST MRI for accurate diagnosis and treatment follow-up in light of the new molecular classification of non-enhancing glioma.

## Supplementary Information

Below is the link to the electronic supplementary material.

Electronic supplementary material 1 (DOCX 85 kb)

## References

[1] Choi YS, Ahn SS, Lee S-K, Chang JH, Kang S-G, Kim SH, Zhou J (2017). Amide proton transfer imaging to discriminate between low- and high-grade gliomas: added value to apparent diffusion coefficient and relative cerebral blood volume. Eur Radiol.

[2] Sakata A, Okada T, Yamamoto A, Kanagaki M, Fushimi Y, Okada T, Dodo T, Arakawa Y, Schmitt B, Miyamoto S, Togashi K (2015). Grading glial tumors with amide proton transfer MR imaging: different analytical approaches. J Neurooncol.

[3] Zhou J, Tryggestad E, Wen Z, Lal B, Zhou T, Grossman R, Wang S, Yan K, Fu D-X, Ford E, Tyler B, Blakeley J, Laterra J, van Zijl PCM (2011). Differentiation between glioma and radiation necrosis using molecular magnetic resonance imaging of endogenous proteins and peptides. Nat Med.

[4] Zou T, Yu H, Jiang C, Wang X, Jiang S, Rui Q, Mei Y, Zhou J, Wen Z (2018). Differentiating the histologic grades of gliomas preoperatively using amide proton transfer-weighted (APTW) and intravoxel incoherent motion MRI. NMR Biomed.

[5] Aum DJ, Kim DH, Beaumont TL, Leuthardt EC, Dunn GP, Kim AH (2014). Molecular and cellular heterogeneity: the hallmark of glioblastoma. Neurosurg Focus.

[6] Louis DN, Perry A, Reifenberger G, von Deimling A, Figarella-Branger D, Cavenee WK, Ohgaki H, Wiestler OD, Kleihues P, Ellison DW (2016). The 2016 World Health Organization Classification of Tumors of the Central Nervous System: a summary. Acta Neuropathol.

[7] Olar A, Wani KM, Alfaro-Munoz KD, Heathcock LE, van Thuijl HF, Gilbert MR, Armstrong TS, Sulman EP, Cahill DP, Vera-Bolanos E, Yuan Y, Reijneveld JC, Ylstra B, Wesseling P, Aldape KD (2015). IDH mutation status and role of WHO grade and mitotic index in overall survival in grade II-III diffuse gliomas. Acta Neuropathol.

[8] Togao O, Hiwatashi A, Yamashita K, Kikuchi K, Keupp J, Yoshimoto K, Kuga D, Yoneyama M, Suzuki SO, Iwaki T, Takahashi M, Iihara K, Honda H (2017). Grading diffuse gliomas without intense contrast enhancement by amide proton transfer MR imaging: comparisons with diffusion- and perfusion-weighted imaging. Eur Radiol.

[9] Jiang S, Zou T, Eberhart CG, Villalobos MAV, Heo H-Y, Zhang Y, Wang Y, Wang X, Yu H, Du Y, van Zijl PCM, Wen Z, Zhou J (2017). Predicting IDH mutation status in grade II gliomas using amide proton transfer-weighted (APTw) MRI. Magn Reson Med.

[10] Jones CK, Polders D, Hua J, Zhu H, Hoogduin HJ, Zhou J, Luijten P, Van Zijl PCM (2012). In vivo three-dimensional whole-brain pulsed steady-state chemical exchange saturation transfer at 7 T. Magn Reson Med.

[11] Deshmane A, Zaiss M, Lindig T, Herz K, Schuppert M, Gandhi C, Bender B, Ernemann U, Scheffler K (2019). 3D gradient echo snapshot CEST MRI with low power saturation for human studies at 3T. Magn Reson Med.

[12] van Zijl PCM, Lam WW, Xu J, Knutsson L, Stanisz GJ (2018). Magnetization transfer contrast and chemical exchange saturation transfer MRI. Features and analysis of the field-dependent saturation spectrum. Neuroimage.

[13] Paech D, Windschuh J, Oberhollenzer J, Dreher C, Sahm F, Meissner JE, Goerke S, Schuenke P, Zaiss M, Regnery S, Bickelhaupt S, Bäumer P, Bendszus M, Wick W, Unterberg A, Bachert P, Ladd ME, Schlemmer HP, Radbruch A (2018). Assessing the predictability of IDH mutation and MGMT methylation status in glioma patients using relaxation-compensated multipool CEST MRI at 7.0 T. Neuro Oncol.

[14] Regnery S, Adeberg S, Dreher C, Oberhollenzer J, Meissner JE, Goerke S, Windschuh J, Deike-Hofmann K, Bickelhaupt S, Zaiss M, Radbruch A, Bendszus M, Wick W, Unterberg A, Rieken S, Debus J, Bachert P, Ladd M, Schlemmer HP, Paech D (2018). Chemical exchange saturation transfer MRI serves as predictor of early progression in glioblastoma patients. Oncotarget.

[15] Heo HY, Jones CK, Hua J, Yadav N, Agarwal S, Zhou J, van Zijl PCM, Pillai JJ (2016). Whole-brain amide proton transfer (APT) and nuclear overhauser enhancement (NOE) imaging in glioma patients using low-power steady-state pulsed chemical exchange saturation transfer (CEST) imaging at 7T. J Magn Reson Imaging.

[16] Block W, Pauly J, Kerr A, Nishimura D (1997). Consistent fat suppression with compensated spectral-spatial pulses. Magn Reson Med.

[17] Zhou J (2011). Amide proton transfer imaging of the human brain. Methods Mol Biol.

[18] Scheidegger R, Wong ET, Alsop DC (2014). Contributors to contrast between glioma and brain tissue in chemical exchange saturation transfer sensitive imaging at 3 Tesla. Neuroimage.

[19] Jones CK, Huang A, Xu J, Edden RAE, Schär M, Hua J, Oskolkov N, Zacà D, Zhou J, McMahon MT, Pillai JJ, van Zijl PCM (2013). Nuclear Overhauser enhancement (NOE) imaging in the human brain at 7T. Neuroimage.

[20] Kuroda J, Kinoshita M, Tanaka H, Nishida T, Nakamura H, Watanabe Y, Tomiyama N, Fujinaka T, Yoshimine T (2012). Cardiac cycle-related volume change in unruptured cerebral aneurysms: a detailed volume quantification study using 4-dimensional CT angiography. Stroke.

[21] Windschuh J, Zaiss M, Meissner JE, Paech D, Radbruch A, Ladd ME, Bachert P (2015). Correction of B1-inhomogeneities for relaxation-compensated CEST imaging at 7T. NMR Biomed.

[22] Yushkevich PA, Piven J, Hazlett HC, Smith RG, Ho S, Gee JC, Gerig G (2006). User-guided 3D active contour segmentation of anatomical structures: Significantly improved efficiency and reliability. Neuroimage.

[23] Jiang S, Eberhart CG, Zhang Y, Heo H-Y, Wen Z, Blair L, Qin H, Lim M, Quinones-Hinojosa A, Weingart JD, Barker PB, Pomper MG, Laterra J, van Zijl PCM, Blakeley JO, Zhou J (2017). Amide proton transfer-weighted magnetic resonance image-guided stereotactic biopsy in patients with newly diagnosed gliomas. Eur J Cancer.

[24] Jiang S, Eberhart CG, Lim M, Heo H-Y, Zhang Y, Blair L, Wen Z, Holdhoff M, Lin D, Huang P, Qin H, Quinones-Hinojosa A, Weingart JD, Barker PB, Pomper MG, Laterra J, van Zijl PCM, Blakeley JO, Zhou J (2019). Identifying recurrent malignant glioma after treatment using amide proton transfer-weighted MR imaging: a validation study with image-guided stereotactic biopsy. Clin cancer Res an Off J Am Assoc Cancer Res.

[25] Yan K, Fu Z, Yang C, Zhang K, Jiang S, Lee DH, Heo HY, Zhang Y, Cole RN, Van Eyk JE, Zhou J (2015). Assessing amide proton transfer (APT) MRI contrast origins in 9 L gliosarcoma in the rat brain using proteomic analysis. Mol Imaging Biol.

[26] Zaiss M, Schuppert M, Deshmane A, Herz K, Ehses P, Füllbier L, Lindig T, Bender B, Ernemann U, Scheffler K (2018). Chemical exchange saturation transfer MRI contrast in the human brain at 9.4 T. Neuroimage.

[27] Harris RJ, Cloughesy TF, Liau LM, Prins RM, Antonios JP, Li D, Yong WH, Pope WB, Lai A, Nghiemphu PL, Ellingson BM (2015). PH-weighted molecular imaging of gliomas using amine chemical exchange saturation transfer MRI. Neuro Oncol.

[28] Xu J, Zaiss M, Zu Z, Li H, Xie J, Gochberg DF, Bachert P, Gore JC (2014). On the origins of chemical exchange saturation transfer (CEST) contrast in tumors at 9.4T. NMR Biomed.

[29] Heo HY, Zhang Y, Lee DH, Hong X, Zhou J (2016). Quantitative assessment of amide proton transfer (APT) and nuclear overhauser enhancement (NOE) imaging with extrapolated semi-solid magnetization transfer reference (EMR) signals: application to a rat glioma model at 4.7 tesla. Magn Reson Med.

[30] Zhou J, Heo HY, Knutsson L, van Zijl PCM, Jiang S (2019). APT-weighted MRI: Techniques, current neuro applications, and challenging issues. J Magn Reson Imaging.

[31] Kim M, Gillen J, Landman BA, Zhou J, Van Zijl PCM (2009). Water saturation shift referencing (WASSR) for chemical exchange saturation transfer (CEST) experiments. Magn Reson Med.

