# Supplementary material for: Mapping tumour heterogeneity with pulsed 3D CEST MRI in non-enhancing glioma at 3 T
Source: MAGMA. 2021 Feb 19;35(1):53–62. doi: 10.1007/s10334-021-00911-6 (PMC8901516; doi:10.1007/s10334-021-00911-6)
Supplement: Supplementary file 1 — Electronic supplementary material 1 (DOCX 85 kb) [file 10334_2021_911_MOESM1_ESM.docx]

**Supplementary information**

*Investigating differences in CEST contrasts and hyperintense volumes per tumour type*

To investigate whether there were significant differences between ROI averaged values for the CEST contrasts calculated between the three different tumour types included in the cohort a linear regression was done that showed no significant effects for either MTR_asym_ (p = 0.465), LD_amide_ (p=0.283) nor LD_NOE_ (p=0.801)_._ Further investigating whether there was an effect of IDH mutation status on either of the contrasts found, we pooled the data for the patients diagnosed with oligodendroglioma and astrocytoma together (N = 12) and used unpaired t-tests to compare this group to the IDH-wildtype patients (N=6). Again, no significant differences were found for either MTR_asym_ (p = 0.668), LD_amide_ (p=0.229) nor LD_NOE_ (p=0.696).

The same analysis was done to compare the hyperintense volumes, and no significant effects were found either (see Figure S1).

| 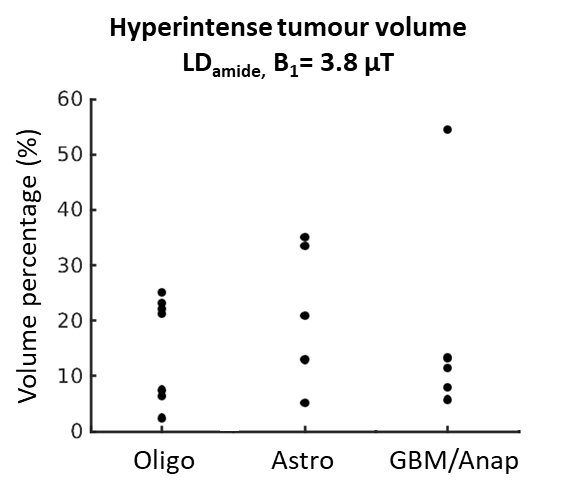Figure S1. Results from calculating hyperintense regions within the tumour ROI per patient. Note that the patients are grouped according to their diagnosis as stated in Table 1, and that the oligodendroglioma (oligo) and astrocytoma (astro) groups were IDH-mutated while the patients in the glioblastoma and anaplastic astrocytoma (GBM/Anap) group showed the IDH-wildtype genotype. The tumour hyperintense ROI includes the voxels that surpass the threshold of LD­­_amide_ based on the average of NAWM LD_amide­_. The volume percentages are plotted as the tumour ROI with hyperintense LD_amide_ signal as a percentage of the whole tumour volume as determined as the hyperintense region on the T_2_-weighted FLAIR. Linear regression showed no significant effect of ‘tumour type’ on these volume percentages (p = 0.730). There was neither a significant difference between IDH-mutation tumours (Oligo + Astro, N = 12) and IDH-wildtype tumours (GBM/Anap, N = 6) (unpaired t-test, p = 0.971). |
| --- |

As indicated in the main manuscript, likely reasons for not corroborating earlier findings of APT-weighted CEST imaging being correlated to tumour grade [*1*,*2*] include the small cohort of only patients diagnosed with non-enhancing glioma included in this study.

**References**

1. Paech D, Windschuh J, Oberhollenzer J, et al. Assessing the predictability of IDH mutation and MGMT methylation status in glioma patients using relaxation-compensated multipool CEST MRI at 7.0 T. *Neuro Oncol*. 2018;20(12):1661-1671.

2. Jiang S, Zou T, Eberhart CG, et al. Predicting IDH mutation status in grade II gliomas using amide proton transfer-weighted (APTw) MRI. *Magn Reson Med*. 2017.
